# Capsule endoscopy findings reflect the gastrointestinal conditions of patients with systemic sclerosis

**DOI:** 10.1038/s41598-021-99775-y

**Published:** 2021-10-11

**Authors:** Sumio Iio, Shiro Oka, Shinji Tanaka, Akihiko Sumioka, Akiyoshi Tsuboi, Takaki Nojima, Shintaro Hirata, Yoshimi Matsuo, Eiji Sugiyama, Michihiro Hide, Koji Arihiro, Kazuaki Chayama

**Affiliations:** 1 Department of Gastroenterology and Metabolism, Hiroshima University Hospital, 1-2-3 Kasumi, Minami-ku, Hiroshima, 734-8551 Japan; 2 Department of Endoscopy, Hiroshima University Hospital, Hiroshima, Japan; 3 Department of Clinical Immunology and Rheumatology, Hiroshima University Hospital, Hiroshima, Japan; 4 Department of Dermatology, Hiroshima University Hospital, Hiroshima, Japan; 5 Department of Anatomical Pathology, Hiroshima University Hospital, Hiroshima, Japan

**Keywords:** Systemic sclerosis, Intestinal diseases

## Abstract

Systemic sclerosis (SSc) is characterized by fibrosis of the skin and various internal organs. However, there is limited knowledge concerning small-bowel lesions. We evaluated the clinical state of patients with SSc according to the capsule endoscopy (CE) findings. Sixty-five consecutive patients with SSc (61 females; mean age, 64.3 years) underwent CE at Hiroshima University Hospital between April 2012 and December 2019. SSc was subclassified into diffuse and limited cutaneous SSc. Among the 65 patients, 55 (51 females; mean age, 64.5 years; diffuse cutaneous SSc, 27 patients) were evaluated for the presence of fibrosis in the gastrointestinal tract by biopsy. Small-bowel lesions were detected in 27 (42%) patients with SSc. Type 1b angioectasia (Yano-Yamamoto classification) was more frequent in limited cutaneous SSc patients (p = 0.0071). The average capsule transit time of the esophagus was significantly longer in diffuse cutaneous SSc patients (p = 0.0418). There were more cases of Type 1a angioectasia in SSc patients without fibrosis. The average capsule transit time of the esophagus was significantly longer in SSc patients with fibrosis. Thus, this study revealed that the frequency of small-bowel angioectasia and gastrointestinal motility in patients with SSc differed depending on SSc subclassification and the presence of fibrosis.

## Introduction

Systemic sclerosis (SSc) is an autoimmune disease characterized by fibrosis of the skin and various internal organs, particularly the gastrointestinal tract, where lesions may lead to motor activity impairment. The degree of sclerosis and the course of progression vary in patients with SSc; SSc can be divided into two subclassifications as follows: diffuse cutaneous SSc (dcSSc) with typical symptoms and limited cutaneous SSc (lcSSc)^1^. Skin sclerosis in dcSSc patients is generalized and often progresses within a few years after the onset of SSc. It is more common when anti-Scl-70 and anti-RNA polymerase III antibodies are detected. In lcSSc patients, the skin sclerosis is often confined to the fingers, within little or no progression, and anti-centromere antibodies are often detected. In dcSSc patients, the capillary of nail and nail fold bleeding are not frequently observed, although capillaroscopy reveals meandering, dilated, tortuous capillaries, and nail fold bleeding.

The gastrointestinal tract is one of the most commonly affected organs in patients with SSc^2,3^. The SSc classification criteria^4^ do not incorporate the gastrointestinal tract manifestations in patients with SSc, although the gastrointestinal tract involvement may result in substantial morbidity and is the most commonly involved organ. Gastrointestinal disease in patients with SSc can manifest as esophageal dysmotility, gastroparesis, colonic dysmotility, and constipation. There are several methods for assessing gastrointestinal motility. These include timed barium esophagram, scintigraphy, and functional magnetic resonance imaging^5^. Scintigraphy is often performed to assess gastrointestinal motility. However, the patients subjected to scintigraphy are exposed to relatively high levels of radiation^6^. On the other hand, capsule endoscopy (CE) allows not only indirect assessment of gastrointestinal motility without radiation exposure but also the detection of small-bowel lesions. The anatomical landmarks identified by CE could be used to calculate the gastrointestinal transit time. Moreover, CE is useful for indirectly examining gastrointestinal motility and detecting small-bowel lesions in patients with digestive symptoms as we reported previously^7^.

This study examined the characteristics of gastrointestinal motility and small-bowel lesions in patients with SSc using CE based on SSc subclassifications and the presence of fibrosis in the gastrointestinal tract.

## Methods

### Patients

We examined the data of 65 consecutive patients with SSc (61 females; mean age, 64 years) who underwent CE at Hiroshima University Hospital between April 2012 and December 2019. Prior to CE, all patients underwent transabdominal ultrasonography to evaluate the gastrointestinal patency^8^. Interestingly, there were no patients with suspected gastrointestinal obstruction in this study.

To evaluate the clinical characteristics in relation to SSc subclassifications, we classified the included patients into two groups according to SSc subclassifications as follows: 31 dcSSc patients (30 females; mean age, 63.4 years) and 34 lcSSc patients (31 females; mean age, 65 years).

All patients included in this study were diagnosed based on the 1980 American College of Rheumatology (ACR) classification criteria for SSc^9^ and/or the 2013 American College of Rheumatology/European league Against Rheumatism (ACR/EULAR) classification criteria for SSc^4^. In addition, all patients were classified as having dcSSc and lcSSc based on the diagnostic criteria reported by LeRoy et al.^1^.

To evaluate the clinical characteristics in relation to the presence of fibrosis in the gastrointestinal tract, 55 patients with SSc (51 females; mean age, 65 years) who provided informed consent and underwent step biopsy from the gastrointestinal tract were evaluated for the presence of fibrosis. Moreover, 10 patients refused to undergo step biopsy after they had informed consent. To assess the presence of fibrosis in the gastrointestinal tract, more than two biopsy samples were taken from each site (esophagus, stomach, duodenum, ileum, colon, and rectum) by upper and lower gastrointestinal endoscopy. Biopsy tissues were evaluated for the presence of fibrosis in the submucosa by a pathologist (K.A).

The study was approved by the Institutional Review Board of Hiroshima University Hospital (approval number: E-1385) and was conducted in accordance with the Declaration of Helsinki. The patients were informed of the risk and benefits of CE at the time of the procedure, and all provided written informed consent to use their de-identified data for research purposes.

### CE procedure

The PillCam™ SB2 or SB3 video capsule (Covidien, Mansfield, MA) was used for CE. The patients swallowed the capsule with a solution of dimeticone after an overnight fast. Sodium picosulfate and magnesium were administered for bowel preparation the night before CE. The patients swallowed the capsule in the sitting position and ate a light meal 4 h after swallowing the capsule. Capsule images were evaluated using the Rapid Reader 6.5 software on a RAPID 8 workstation (Covidien, Mansfield, MA). Two experienced endoscopists, who were experienced in reading more than 200 capsule videos, reviewed and interpreted the CE image stream independently. The diagnosis was reached by consensus.

### Evaluation

We evaluated the patients with SSc concerning the average capsule transit time (esophagus, stomach, and small-bowel), total small-bowel observation rate, frequency and characteristics of small-bowel lesions, and detection rate of small-bowel lesions. Small-bowel vascular lesions were classified according to Yano-Yamamoto classification^10^. Gastrointestinal motility was assessed by measuring the capsule transit time in the gastrointestinal tract (esophagus, stomach, and small bowel). The following characteristics were evaluated: sex, age, SSc subclassification, abdominal surgical history, medication, chief complaint, modified Rodnan’s total skin thickness score (mRSS), telangiectasia, disease duration in relation to SSc subclassification, and the presence of fibrosis in the gastrointestinal tract.

### Statistical analysis

Quantitative variables were compared using Pearson’s chi-square or Fischer’s exact test, whereas continuous variables were compared using Student’s t-test or the Mann–Whitney U test. All tests were two-sided, and *p*-values < 0.05 were considered statistically significant. Differences between the subgroups were examined using the log-rank test. All analyses were performed using JMP version 15 (SAS Institute Inc., Cary, NC).

## Results

**Figure 1:**
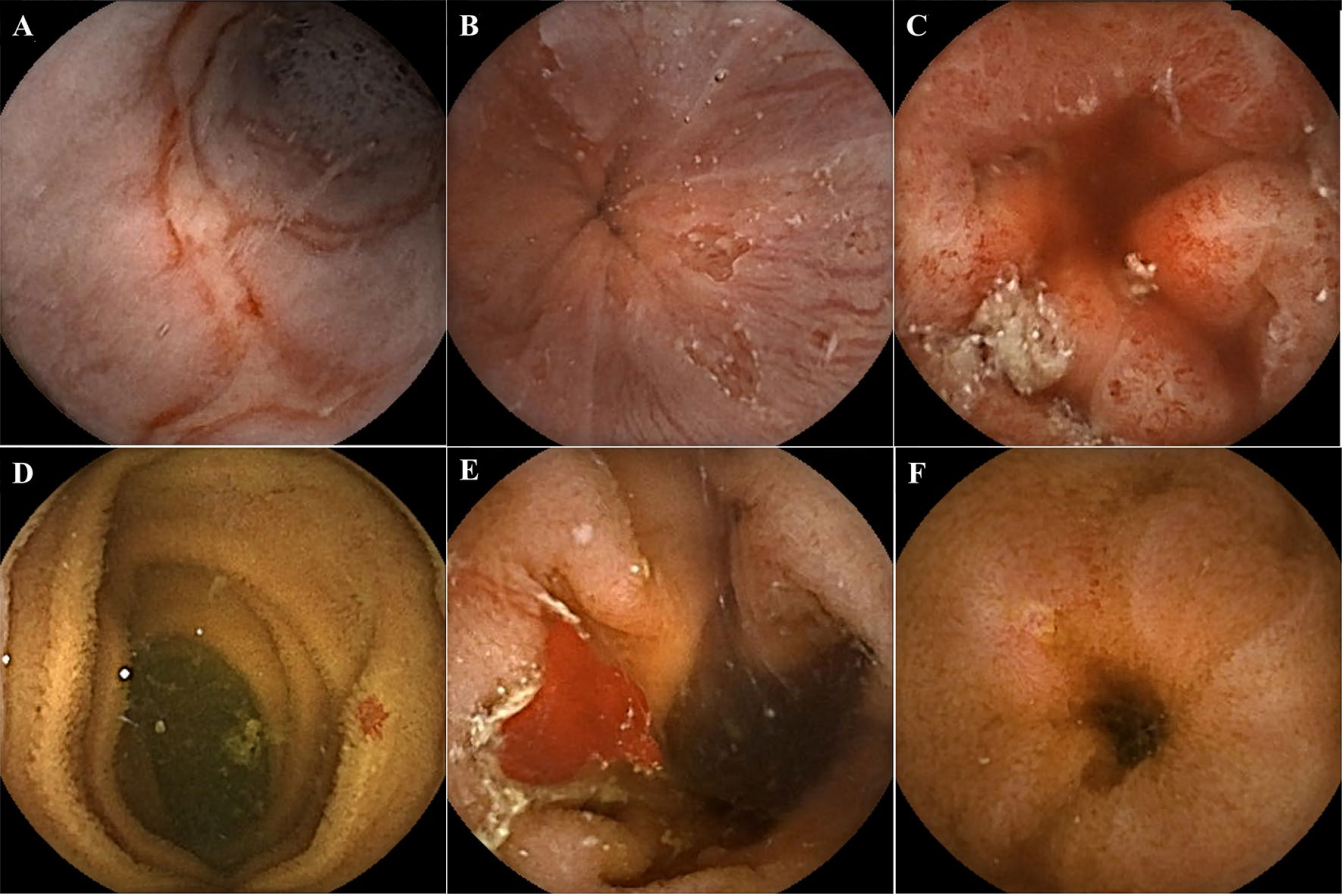
Gastrointestinal lesions identified by CE. (**A**) Reflux esophagitis, (**B**) SSBE, (**C**) GAVE, (**D**) Type 1b angioectasia, (**E**) Angioectasia with oozing (**F**) Erosion. CE: capsule endoscopy, SSBE: short segment Barrett’s esophagus, GAVE: gastric antral vascular ectasia.

Table 1 shows the characteristics of the two groups. Both groups included more female than male patients, with similar ratios (dcSSc: 97% [30/31] females; lcSSc: 91% [31/34] females; p = 0.3363). Moreover, there was no significant difference in age (dcSSc: 63.4 [36–80] years; lcSSc: 65.2 [37–81] years; p = 0.2663). None of the patients in both groups had a history of abdominal surgery. There was no significant difference in the use of non-steroidal anti-inflammatory drugs (NSAIDs) or anti-platelet drugs, chief complaints, the presence of telangiectasia, or disease duration. Anti-platelet drugs were used in three dcSSc patients and five lcSSc patients (p = 0.5353), whereas NSAIDs were used in three dcSSc patients and seven lcSSc patients (p = 0.2168). Regarding chief complaints, 50% (33/63) of patients with SSc were asymptomatic. There was a significant difference in mRSS. This is consistent with the disease backgrounds. The average capsule transit time of the esophagus was significantly longer in dcSSc patients than in lcSSc patients (16.0 vs. 3.8 min, p = 0.0418) (Table 2). The average capsule transit time in the stomach and small bowel was not significantly different between the two groups (stomach: 35.2 vs. 36.4 min, p = 0.1904; small bowel: 268.5 vs. 263.7 min, p = 0.8412). There was no significant difference between the two groups in the total small-bowel observation rate (65% [20/31] vs. 71% [24/34]). The frequency and characteristics of gastrointestinal lesions are shown in Table 2. Small-bowel lesions were detected in 42% (27/65) of patients with SSc; 13 patients (20%) had angioectasia, and 14 (22%) patients had erosion/ulceration. Ten patients with SSc (15%) had reflux esophagitis, and five (8%) had short-segment Barrett’s esophagus. Five patients with SSc were found to have gastric antral vascular ectasia. Gastrointestinal lesions identified by CE are presented in Fig. 1. Small-bowel lesions, especially Type 1b angioectasia, were significantly more frequent in lcSSc patients (17%, 6/34) than in dcSSc patients (0%, 0/31). Of the seven patients with Type 1a angioectasia, four were asymptomatic, and three had anemia. All six patients with Type 1b angioectasia had anemia (mean hemoglobin level, 8.6 [6.5–10.3] g/dL).

**Table 1:** Characteristics of patients with systemic sclerosis at the time of capsule endoscopy.

Variables	Total, n = 65	Types		*P*-values
		dcSSc, n = 31	lcSSc, n = 34	
Female	61 (94)	30 (97)	31 (91)	0.3363
Mean age (y. o.)	64.3 [36–81]	63.4 [36–80]	65.2 [37–81]	0.2663
Abdominal surgery history (+)	0 (0)	0 (0)	0 (0)	
**Medication**				
Anti-platelet drugs	8 (12)	3 (10)	5 (15)	0.5353
NSAIDs	10 (15)	3 (10)	7 (21)	0.2168
**Chief complaint**				
Abdominal symptoms	18 (28)	8 (26)	10 (29)	
OGIB	14 (22)	6 (19)	8 (24)	
No symptoms	33 (50)	17 (55)	16 (47)	
mRSS	11.1 [0–43]	16.5 [0–43]	7.3 [0–12]	0.0015
Telangiectasia	18 (28)	9 (29)	9 (26)	0.8177
Disease duration (month)	64.1 [7–192]	53.5 [7–131]	73.8 [8–192]	0.0598

Patients were stratified according to the SSc subclassification. Data are shown as frequencies (percentages) or means, as appropriate.

SSc: systemic sclerosis, dcSSc: diffuse cutaneous SSc, lcSSc: limited cutaneous SSc, NSAIDs: non-steroidal anti-inflammatory drug, OGIB: obscure gastrointestinal bleeding, mRSS: modified Rodnan’s total skin thickness score.

**Table 2:** The average capsule transit time and gastrointestinal lesions in patients with systemic sclerosis.

Variables	Total, n = 65	Types		*P*-values
		dcSSc, n = 31	lcSSc, n = 34	
Total small-bowel observation rate	68% (44/65)	65% (20/31)	71% (24/34)	0.6011
**Transit time (min)**				
Esophagus	12.6 [1–238]	16.0 [1–238]	3.8 [1–29]	0.0418
Stomach	35.8 [1–175]	35.2 [1–175]	36.4 [1–147]	0.1904
Small bowel	265.9 [136–430]	268.5 [136–428]	263.7 [145–430]	0.8412
**Gastrointestinal lesions**				
Esophagus				
Reflux esophagitis	10 (15)	4 (13)	6 (17)	0.5951
SSBE	5 (8)	2 (6)	3 (9)	0.7189
Stomach				
GAVE	5 (8)	4 (13)	1 (3)	0.1221
Small-bowel				
Angioectasia	13 (20)	4 (13)	9 (26)	0.0797
Type 1a	7 (11)	4 (13)	3 (× 9)	0.7859
Type 1b	6 (9)	0 (0)	6 (17)	0.0071
Erosion/ulceration	14 (22)	9 (29)	5 (15)	0.1589

Data are shown as frequencies (percentages) or means, as appropriate.

SSc: systemic sclerosis, dcSSc: diffuse cutaneous SSc, lcSSc: limited cutaneous SSc, SSBE: short-segment Barret’s esophagus, GAVE: gastric antral vascular ectasia.

Table 3 shows treatment methods for small-bowel lesions in patients with SSc. Type 1a angioectasia without oozing was followed up without any treatment. Endoscopic hemostasis was performed in patients with oozing Type 1a angioectasia (polidocanol injection (PDI) alone) and Type 1b angioectasia (PDI combined with argon plasma coagulation or clipping) as we previously reported the usefulness of PDI for small-bowel angioectasia^11^. Patients with erosion/ulceration were treated internally.

**Table 3:** Treatment methods for small-bowel lesions in patients with systemic sclerosis.

dcSSc, n = 31		lcSSc, n = 34	
Angioectasia	4 (13)	Angioectasia	9 (26)
**Type 1a**		**Type 1a**	
Endoscopic hemostasis (PDI)	4 (13)	Endoscopic hemostasis (PDI)	3 (9)
**Erosion/ulceration**	9 (29)	**Type 1b**	
Medication	9 (29)	Endoscopic hemostasis (PDI combined with APC or clipping)	6 (17)
		**Erosion/ulceration**	5 (15)
		Medication	5 (15)

Data are shown as frequencies (percentages) or means, as appropriate.

SSc: systemic sclerosis, dcSSc: diffuse cutaneous SSc, lcSSc: limited cutaneous SSc, PDI: polidocanol injection, APC: argon plasma coagulation.

Among 55 patients with SSc who underwent step biopsies, 17 patients (14 females; mean age, 69 years) had fibrosis. There was no significant difference in sex, percentage of subclassification, mean age, medication, chief complaint, mRSS, the presence of telangiectasia, or disease duration (Table 4). The average transit time in the esophagus was significantly longer in patients with fibrosis than in those without fibrosis (28.8 vs. 5.5 min, p = 0.0448). The average transit time in the stomach and small-bowel showed no significant differences between the two groups (stomach: 26.8 vs. 41.9 min, p = 0.0959; small-bowel: 272.5 vs. 260.3 min, p = 0.6590). The total observation rate of the small bowel was not significantly different between the two groups (fibrosis (+): 71% [12/17] vs. fibrosis (−): 71% [27/38], p = 0.9721) (Table 5). Angioectasia was more frequent in SSc patients without fibrosis (11/38, 29%) than in those with fibrosis (1/17, 9%) (p = 0.0365). In addition, Type 1a angioectasia was significantly more frequent in patients without fibrosis (7/38, 18%) than in those with fibrosis (0/17, 0%) (p = 0.177). There was no significant difference in the frequency of small-bowel lesions between the two groups (Table 5). CE was performed without causing adverse events in all patients.

**Table 4:** Patient characteristics according to the presence of fibrosis in the gastrointestinal tract.

Variables	Fibrosis		*P*-values
	(+), n = 17	(−), n = 38	
Female	14 (82)	37 (97)	0.0586
Subclassification (dcSSc)	10 (59)	17 (45)	0.3333
Mean age (y. o.)	68.7 [58–81]	62.7 [36–80]	0.3568
**Medication**			
Anti-platelet drugs	3 (18)	4 (11)	0.3486
NSAIDs	2 (12)	6 (16)	0.6771
**Chief complaint**			
Abdominal symptoms	5 (29)	10 (26)	
OGIB	2 (12)	11 (29)	
No symptoms	10 (59)	17 (45)	
mRSS	13.9 [0–43]	9.4 [0–34]	0.1623
Telangiectasia	5 (29)	10 (26)	0.8117
Disease duration (month)	56.6 [10–120]	65.8 [7–192]	0.4783

Data are shown as frequencies (percentages) or means, as appropriate.

SSc: systemic sclerosis, dcSSc: diffuse cutaneous SSc, NSAIDs: non-steroidal anti-inflammatory drug, OGIB: obscure gastrointestinal bleeding, mRSS: modified Rodnan’s total skin thickness score.

**Table 5:** The average capsule transit time and small-bowel lesions according to the presence of fibrosis in the gastrointestinal tract.

Variables	Fibrosis		*P*-values
	(+), n = 17	(−), n = 38	
Total small-bowel observation rate	71% (12/17)	71% (27/38)	0.9721
**Transit time (min)**			
Esophagus	28.8 [1–238]	5.5 [1–135]	0.0448
Stomach	26.8 [1–147]	41.9 [1–175]	0.0959
Small bowel	272.5 [156–359]	260.3 [136–430]	0.6590
**Small-bowel lesions**			
Angioectasia	1 (9)	11 (29)	0.0365
Type 1a	0 (0)	7 (18)	0.0177
Type 1b	1 (9)	4 (11)	0.5657
Erosion/ulceration	5 (45)	6 (14)	0.2544
Total	6 (54)	17 (43)	0.5095

Data are shown as frequencies (percentages) or means, as appropriate.

## Discussion

Our study revealed a high frequency of small-bowel lesions (42%) in patients with SSc, which is almost identical to the frequency of small-bowel lesions reported by Marie et al. (52%)^12^. Previous studies have not examined detailed gastrointestinal lesions based on the SSc subclassification and the presence of fibrosis. However, SSc cases may vary greatly in severity and clinical course; gastrointestinal lesions may also differ according to SSc subclassifications. Our study showed that lcSSc patients had a higher frequency of Type 1b angioectasia than dcSSc patients. It is known that lcSSc patients have more telangiectasia and bleeding points in the nail epithelium than dcSSc patients^1,4^, and the frequency of angioectasia is thought to be higher in lcSSc patients. The frequency of angioectasia (especially Type 1a angioectasia) in the small bowel was significantly higher in the gastrointestinal tract in the group with fibrosis, suggesting that the frequency of small-bowel lesions in patients with SSc is associated with fibrosis of the gastrointestinal tract.

Small-bowel angioectasia is considered to cause obscure gastrointestinal bleeding^13,14^ and is common in patients with underlying conditions, such as aortic stenosis and chronic obstructive pulmonary disease^15–18^. It is thought to be an acquired vascular lesion caused by chronic hypoxia in the microcirculation^19–21^, which may require appropriate treatment^11,22^. If small-bowel lesions, especially small-bowel angioectasia indicated for treatment (Type 1a with oozing/Type 1b), are left untreated, they can cause anemia. Screening with CE may be able to detect small-bowel lesions at an early stage. Therefore, patients with SSc are recommended to undergo screening of the small-bowel at least once, and if small-bowel lesions, such as small-bowel angioectasia are detected, endoscopic treatment should be performed. Moreover, patients with small-bowel angioectasia should be followed up for at least 1 year^11^.

The capsule transit time in the gastrointestinal tract can be used to indirectly evaluate gastrointestinal motility, although it does not accurately reflect gastrointestinal motility. In patients with SSc, the average capsule transit time in the esophagus was 12.6 min, which is much longer than that in healthy people^23^. This study revealed a significantly longer transit time in the esophagus, especially in dcSSc patients and those with fibrosis. In patients with SSc, the deposition of collagen fibers in the submucosa and muscularis propria causes muscle tissue rupture and atrophy, which may lead to decreased peristalsis in the gastrointestinal tract. Especially, dcSSc patients present with gastrointestinal symptoms and have more severe symptoms earlier than lcSSc patients^1,4^. This is thought to be related to the longer capsule transit time in the esophagus. It has been reported that SSc is associated with diseases derived from gastrointestinal dysfunction, including small intestine bacterial overgrowth^24,25^. To our knowledge, most studies, to date, have focused on patients with advanced-stage SSc^26–28^, but only a few have reported on SSc patients with mild symptoms, such as those included in this study. Our findings suggest that CE is useful in assessing the clinical condition of patients with SSc, even in asymptomatic SSc patients.

An advantage of CE is the ability to evaluate the motility of the gastrointestinal tract indirectly without the need for radiation exposure or drug administration during the examination^29^; moreover, the measurement of the capsule transit time may be useful in assessing gastrointestinal function in patients with SSc. It has been reported that SSc disproportionately affects the upper tract, and up to 90% of patients have dysmotility, as inferred by the radionuclide transit time^30^. Up to 90% of patients experience upper and/or lower gastrointestinal dysmotility symptoms, which may be associated with increased morbidity and mortality rates^31^. The true prevalence of gastrointestinal involvement remains unknown; however, it is reported that up to 70% of patients take medications that specifically address gastrointestinal symptoms^32^. The severity of the gastrointestinal disease has also been shown to be a marker for worse prognosis and mortality in patients with SSc^33–37^. In this study, the capsule transit time in the esophagus was significantly longer in patients with fibrosis; therefore, the presence of fibrosis might have affected the capsule transit time. There was no difference in clinical symptoms between patients with and without fibrosis, and most patients were asymptomatic. CE might be a clue to the decrease in esophageal peristalsis. The esophageal transit time indirectly reflect the esophageal motility and is less accurate than high-resolution manometry. High-resolution manometry may be a good indication for patients with suspected esophageal motility disorder. However, CE can be performed more easily than high-resolution manometry except for patients with dysphagia or gastrointestinal stenosis. In addition to indirectly assessing gastrointestinal motility, CE also allows examination of the gastrointestinal tract.

Our study had some limitations. First, it was a retrospective analysis and thus the selection bias could not be avoided. Second, the sample size was relatively small. Third, the data were obtained from a single-center, and our observation period was short. Fourth, CE was performed only once for each patient. Therefore, surveillance CE is necessary for SSc patients with CE findings. Finally, step biopsy was not performed in all patients. Therefore, further large-scale studies are needed to address these limitations.

In conclusion, CE is a useful modality for the identifing small-bowel lesions and diagnosis of angioectasia in lcSSc patients. In addition, the capsule transit time in the esophagus could be useful in evaluating the clinical condition of patients with SSc.

## Data Availability

The data that support the findings of this study are available from the corresponding author (S.O) upon reasonable request.

## Abbreviations

SSc: Systemic sclerosis
dcSSc: Diffuse cutaneous SSc
lcSSc: Limited cutaneous SSc
CE: Capsule endoscopy
mRSS: Modified Rodnan’s total skin thickness score
NSAIDs: Non-steroidal anti-inflammatory drug
PDI: Polidocanol injection
APC: Argon plasma coagulation
OGIB: Obscure gastrointestinal bleeding
SSBE: Short-segment Barrett’s esophagus
GAVE: Gastric antral vascular ectasia
